# CXCR4 and its ligand CXCL12 display opposite expression profiles in feline mammary metastatic disease, with the exception of HER2-overexpressing tumors

**DOI:** 10.1186/s12885-018-4650-9

**Published:** 2018-07-16

**Authors:** Cláudia S. Marques, Ana Rita Santos, Andreia Gameiro, Jorge Correia, Fernando Ferreira

**Affiliations:** Center for Interdisciplinary Research in Animal Health, Faculty of Veterinary Medicine, University of Lisbon, 1300-477 Lisbon, Portugal

**Keywords:** Feline mammary carcinoma, CXCR4, CXCL12, Primary tumors, Metastases, Human breast cancer model

## Abstract

**Background:**

The receptor CXCR4 and its ligand CXCL12 play crucial roles in breast cancer. Despite the fact that the spontaneous feline mammary carcinoma (FMC) is considered a suitable model for breast cancer studies, the importance of the CXCR4/CXCL12 axis in FMC is completely unknown. Therefore, this work aims to elucidate the role of CXCR4 and its ligand in the progression of FMC and metastatic disease.

**Methods:**

CXCR4 and CXCL12 expression was analyzed by immunohistochemistry and immunofluorescence on primary tumors (PT), regional and distant metastases of female cats with mammary carcinoma and correlated with serum CXCL12 levels, tumor molecular subtypes and clinicopathological features.

**Results:**

CXCR4 was more expressed in PT than in metastases (*p* = 0.0067), whereas CXCL12 was highly expressed in metastatic lesions located in liver and lung (*p* < 0.0001), as reported for human breast cancer. Moreover, cats with CXCR4 positive PT exhibited significantly lower serum CXCL12 levels than cats with CXCR4 negative mammary carcinomas (*p* = 0.0324). At metastatic lesions, HER2-overexpressing tumors presented higher CXCR4 expression than the other molecular tumor subtypes (*p* = 0.012) and significant differences in overall (*p* = 0.0147) and disease-free survival (*p* = 0.0279) curves between the cats with CXCL12 positive and CXCL12 negative tumors were found. Indeed, CXCL12 negative PT were associated with unfavorable prognosis in cats with HER2-overexpressing tumors.

**Conclusions:**

This work exposes part of the complex interaction between CXCR4 and CXCL12 in PT, but also in metastases of a breast cancer model. These findings could uncover novel therapeutic tools to be used in cats and humans.

## Background

The chemokine CXCL12, also known as SDF-1, binds to G-protein-coupled seven-transmembrane-domain chemokine receptor 4 (CXCR4), activating signaling pathways (PI-3 K/AKT, ERK1/2 and MAPK) and controlling cell survival, migration and proliferation, with the CXCR4/CXCL12 axis showing a key role in breast cancer progression and in many other cancers, as in liver, lung, bone, brain, prostate, ovarian, cervical, colorectal and pancreatic tumors [1–4]. Evidence for a regulatory role of CXCR4/CXCL12 axis in the progression of the metastatic disease was found in breast cancer patients [5] with the organs and tissues with highest CXCL12 expression frequently showing metastases and with CXCL12 working as a chemotactic factor. On the other hand, CXCR4 is mainly expressed in primary breast cancer lesions and lymph node metastases [5, 6]. In fact, patients with breast tumors showing CXCR4 overexpressing were associated with an increased number of metastases in lymph nodes and a decreased overall survival comparing to tumors with low CXCR4 expression. On the other hand, the synthesis of CXCL12 by stroma cells may support tumor progression by autocrine and paracrine mechanisms [6–9]. More recently, an extensive amount of research has been conducted to clarify the role of CXCR4/CXCL12 axis in human breast cancer and metastatic disease [1, 2, 10–12], suggesting that targeted therapies against CXCR4/CXCL12 axis may inhibit tumor growth. Indeed, some CXCR4 antagonists have been developed and tested in clinical trials or even approved by the US Food and Drug Administration [13]. In parallel, an anti-human CXCR4 antibody is being tested in phase I trial [14] and an anti-CXCL12 aptamer (NOX-A12, Noxxon Pharma) is in phase I/II trials [15]. Several preclinical studies targeting the CXCR4/CXCL12 axis have been recently conducted in breast tumors. In 2017, a preclinical study showed that two CXCR4 inhibitors (AMD3100 and TN14003) significantly reduced tumor growth in a HER2 overexpressing tumor xenographs, including Herceptin and Docetaxel-resistant, suggesting that CXCR4 inhibition could be a useful strategy for treat HER2 breast cancer patients [16]. In addition, Nef-M1, a CXCR4 antagonist peptide, also showed good therapeutic potential for inhibiting tumor angiogenesis and the oncogenic epithelial-to-mesenchymal transition process in patient derived xenographs [17].

The feline mammary carcinoma (FMC), one of the most common tumor in cats, shares several clinicopathological features with human breast cancer and is considered a suitable model for comparative oncology [18–20]. However, the improvement of the diagnostic and treatment in cats with mammary carcinoma is needed because the disease is characterized by a very poor prognosis [18–20]. So far, data regarding the involvement of the CXCR4/CXCL12 axis in FMC revealed that CXCR4 is overexpressed in the majority of FMC [21–23] with frequent metastization at lymph node, liver and lung as reported in human breast cancer. The proliferative role of CXCR4/CXCL12 axis was also demonstrated in ex vivo feline cells and recently our team found that serum CXCL12 levels can serve as a diagnostic marker of FMC and in particular for HER2-overexpressing tumors [24].

Taking into account the relevant oncogenic role of the CXCR4/CXCL12 axis in breast cancer progression and its potential to be targeted by anti-tumor molecules, we aimed to clarify the signature of CXCR4 and CXCL12 in cats with mammary metastatic disease and search for significant associations between CXCR4 and CXCL12 tissue status and serum CXCL12 values, clinicopathological features and FMC molecular subtypes.

## Methods

### Animal collection

A population of 115 female cats with mammary tumors admitted to the Veterinary Teaching Hospital of the Faculty of Veterinary Medicine (University of Lisbon) from June 2012 to December 2016 was used in this study, after owner’s permission. All mammary and metastatic lesions were excised during surgery or necropsy and embedded in paraffin after fixation in 10% buffered formalin neutralized with 0.1 M phosphate buffer (pH 7.2), for 24–48 h. The presence of regional lymph node metastases was evaluated in 105 cats with 49 axillary and retromammary lymph nodes tissues from 47 queens being assessed. A full postmortem examination was performed and metastatic disease was confirmed histologically with 24 lungs and 7 livers being collected from 20 cats. The following clinical data were collected from each animal: age, breed, reproductive status, administration of progestogens, number and location of tumors, tumor size and stage (TNM system) [25] treatment prescribed (none, mastectomy, mastectomy combined with chemotherapy). The degree of malignancy and histopathologic classification were evaluated [26, 27]. Information about presence of tumor necrosis, lymphatic invasion by tumor cells, lymphocytic infiltration and cutaneous ulceration was collected. Disease-free survival (DFS) and overall survival (OS) were also recorded. Additionally, blood samples were collected from 42 queens with mammary disease. Serum was isolated from clotted blood by centrifugation (1500 g, 10 min, 4 °C) and immediately frozen at − 80 °C. All samples that showed hemolysis were discarded, as recommended for humans [28].

### Assessment of CXCR4, CXCL12, HER2, ER, PR and Ki-67 tissue status by immunohistochemistry (IHC)

IHC was conducted as previously described [18, 24]. Briefly, a representative area of each tumor lesion with a diameter of 6 mm and 3 μm thickness were mounted on Superfrost® plus microscope slides (ThermoFisher Scientific, Waltham, USA). Xylene was used to deparaffinize and an ethanol/water gradient series was used to rehydrate the sections. For CXCL12 immunostaining, tissue slides were immersed in Novocastra™ epitope retrieval solution pH 6 (Leica Biosystems, Wetzlar, Germany) for CXCL12 staining and then boiled in a microwave for antigen retrieval (25 min at 600 W). The staining was performed using the Novolink™ Polymer Detection System, Leica Biosystems, following the manufacturer’s procedure. The following primary human antibodies diluted in Lab Vision™ Antibody Diluent OP Quanto (ThermoFisher Scientific) were used: rabbit monoclonal anti-CXCR4 antibody (clone UMB2, 1:500, Abcam, Cambridge, UK), mouse monoclonal anti-CXCL12α antibody (clone 79,018, 1:50, R&D Systems, Minneapolis, USA), mouse anti-HER2 (clone CB11, 1:200, Invitrogen, Carlsbad, CA, USA), mouse anti-ER (clone 6F11, 1:125, ThermoFisher Scientific), rabbit anti-PR (clone 1E2, ready-to-use, Ventana, Tucson, USA) and rabbit anti-Ki-67 (polyclonal, 1:500, ThermoFisher Scientific). Samples of FMC known to have high CXCR4 and CXCL12 expression and feline tonsil tissue sample were used as positive controls. Tissue sections incubated with no primary antibodies and feline mammary normal samples were used as negative controls. All slides were scored in a blind manner by two independent pathologists and in doubtful and/or divergent IHC results, cases were re-evaluated using a multiobserver microscope and the staining was discussed until a consensus was achieved. Images were taken with an optical microscope system (Axiovert S100 with AxioCam HRc; Carl Zeiss BV, Sliedrecht, the Netherlands) and analyzed using AxioVision (Carl Zeiss).

### Scoring of IHC staining results

The scoring system for CXCR4 was performed as previously described in humans and cats [9, 24, 29, 30]. Briefly, staining intensity of the cell membrane and/or cytoplasm was graded as 0 (negative), 1 (weak), 2 (moderate) and 3 (strong) and the percentage of staining cells was determined by evaluating at least 1000 neoplasic cells in 10 high-power fields (400× magnification) for each tissue section and classified as 0 = negative, 1 = <10%, 2 = 10–50%, and 3 = >50%. To obtain staining indexes, the intensity and percentage scores were multiplied, with staining indexes of 0 and 1 considered CXCR4-negative (0), as the staining indexes 2 and 3 (1+), while staining indexes of 4 and 6 were considered positive (2+), as well as the staining index of 9 (3+). CXCL12 scoring system was based on the percentage of membrane and/or cytoplasm stained tumor cells and their relative staining intensity, as previously described for breast cancer studies [6, 8, 9, 30, 31]. Absence of staining was scored as 0, 1 to 10% of positive cells were scored as 1, 11 to 50% as 2, 51 to 80% as 3, and 81 to 100% as 4. Staining intensity was scored from 0 to 3 as follows: 0 = negative, 1 = weak, 2 = moderate and 3 = strong staining. The percentage and staining intensity scores were multiplied and data was converted to the german immunoreactive score (IRS) ranging between 0 and 12 with samples scoring ≥3 being considered positive for CXCL12 expression. HER2 immunoreactivity was scored according to the American Society of Clinical Oncology’s guidelines. Briefly, FMC were classified as HER2-negative when scored 0 or +1 and HER2-positive if scored as + 2 or + 3. Mammary carcinomas were also evaluated for ER/PR status using the Allred score system, and only tumors with a score ≥2 were considered positive. The Ki-67 proliferation index was determined by dividing the number of tumoral cells showing positive nuclear immunostaining per 1000 tumor cells analyzed over at least three high-amplified microscopic fields. Tumors were considered highly proliferative when more than 14% of the neoplastic cells nuclei expressed Ki-67 [18, 32].

### Tissue CXCR4 and CXCL12 immunofluorescence labeling

Double immunofluorescence labeling was performed in the same tissue samples evaluated in the IHC assay. The non-specific staining was blocked with 0.4% casein in PBS, with stabilizers, surfactant, and 0.2% Bronidox (Novolink™ Protein Block, Leica Biosystems). Tissue samples were double stained overnight at 4 °C, with the following primary antibodies rabbit monoclonal anti-CXCR4 antibody (clone UMB2, 1:500, Abcam) and mouse monoclonal anti-CXCL12α antibody (clone 79,018, 1:50, R&D Systems, Minneapolis, USA), diluted in Lab Vision™ Antibody Diluent OP Quanto (ThermoFisher Scientific). After several washes with PBS, tissue sections were incubated 30 min at room temperature with the secondary antibodies: goat anti-rabbit IgG Alexa Fluor® 594 (Abcam, 1:1000) and donkey anti-mouse IgG H&L Alexa Fluor® 488 (Abcam, 1:500). From this step forward, samples were protected from light in order to prevent fluorochrome fading and the sections washed, at least three times, in PBS during 15 min. Then, one drop of fluoroshield mounting medium with DAPI (Abcam) was applied directly on top of the specimen. Slides were cover slipped, sealed with clear nail varnish and observed in a Leica DMIRE2 epifluorescence microscope (Leica Microsystems) equipped with a CoolSNAP HQ CCD camera (Photometrics, Tucson, AZ, USA). Images in appropriate fluorescence filter sets, corresponding to the signals of DAPI, CXCR4-Alexa Fluor® 594 and CXCL12-Alexa Fluor® 488 were acquired with Photoshop CS5 software (Adobe Systems, Inc., San Jose, USA). Analysis of data sets preparation was performed with the open-source Java-based image processing program software Image J (version 1.51p 22, National Institutes of Health, Bethesda, USA).

### Quantification of serum CXCL12 levels by ELISA

CXCL12 protein serum concentration were evaluated by using a commercial ELISA-based kit (CXCL12/CXCL12 DuoSet ELISA kit, R&D Systems, Minneapolis, USA), following the manufacturer’s protocol and our previous publication [24]. Briefly, for each ELISA assay, a standard curve was generated using seven dilutions of the recombinant CXCL12, with known concentrations. A 96-well ELISA plate was coated overnight with 1 μg/ml of mouse anti-CXCL12 capture antibody (100 μl) diluted in bovine serum albumin - phosphate buffer solution (1% *w*/*v* BSA in PBS). After several washes (0.05% *v*/v Tween-20 in PBS), each well of the plate was blocked (1% w/v BSA in PBS) for 1 h to prevent non-specific binding and 100 μl of diluted serum samples (1:10) or standards dilutions were incubated for 2 h. Then, the plate was washed and 50 ng/ml of the biotinylated goat anti-CXCL12 detection antibody (100 μl) was added to each well for 1 h incubation. Later, the conjugated streptavidin-horseradish peroxidase (HRP) was diluted 40 times and incubated in the plate wells for 45 min after previous washes. A final wash was performed before adding 100ul of the HRP substrate (3,3′,5,5′-tetramethylbenzidine) solution (R&D Systems) during 25 min in the dark. The reaction was stopped with 50 μl of 2 N sulfuric acid. The absorbance was measured in a spectrophotometer (LabSystems IEMS Reader MF, Labsystems/Thermo Scientific, Helsinki, Finland) using 450 nm as the primary wavelength and 570 nm as reference wavelength.

### Statistical analysis

Graphpad Prism version 7.02 (La Jolla, USA) was used for all statistical analysis and *p* values< 0.05 was considered statistically significant. Outliers were removed from analysis based on the combination of **R**obust regression and **Out**lier removal (ROUT method) implemented in Graphpad Prism software. This method identifies outliers when fitting data with nonlinear regression, with reasonable power and few false positives [33]. The non-parametric Mann-Whitney test was used to compare serum CXCL12 levels in CXCR4 or CXCL12 negative vs CXCR4 or CXCL12 positive tumor samples. The Fisher’s exact test was used to assess the differences in CXCR4 or CXCL12 expression rates between PT and metastases and among different FMC molecular subtypes. The association between the expression rates and different clinicopathological features measured in an ordinal or nominal scale (categorical variables) was also evaluated using the Fisher’s exact test. OS and DFS were analyzed by the Kaplan–Meier method (log-rank test).

## Results

### Animal population

A total of 115 female cats with mammary carcinoma and showing a mean age of 11.40 ± 2.82 years, ranging from 5 to 18 years, were enrolled in this study. Their clinicopathological features are summarized in Tables 1 and 2. Ninety seven animals (84.3%) were submitted to surgical mastectomy and 8 (7.0%) were subjected to anthracycline-based adjuvant chemotherapy (doxorubicin, 25 mg/m2, intravenously, every 3 weeks for 5 cycles). Forty-five percent (*n* = 52) of the PT were at stage III (45.2%), being frequently classified as tubulopapillary carcinomas (37/115; 32.2%) or tubular carcinoma (30/115; 26.1%) and showing a high-grade of malignancy (91/115, 79.1%). Regarding molecular markers, 32 animals (27.8%) showed HER2-overexpressing mammary carcinomas, 49 cats had PR-positive (42.6%) and 29 (25.2%) ER-positive tumors. Eighty-three animals (72.2%) presented FMC with a high Ki-67 index. The overall survival (OS) was 12.94 ± 10.28 months and the survival ratio was 50.43%. The disease free-survival (DFS) was 8.78 ± 7.85 months and 64 of the cats (55.65%) had disease recurrence until the end of the follow-up period (54 months). Among these animals, 44 (38.3%) showed a local relapse (Table 1), 47 (73.4%) regional metastases, and 20 (31.2%) had distant metastases (Table 2). From the 47 animals with regional metastatic disease, 18 (38.3%) presented metastases at the axillary lymph nodes and 29 (61.7%) at retromammary lymph nodes (Table 2). Lung metastases were found in 20 cats whereas seven animals presented both lung and liver metastases (Table 2). Finally, 14 out of 47 animals (29.8%) showed HER2-overexpressing RM whereas no HER2-overexpressing were found in DM (*n* = 20). Twenty-three RM and 7 DM were stained as PR-positive, while 15 RM and 6 DM were ER-positive. Forty-one animals with RM, 13 lung and 5 liver metastases, presented a high Ki-67 index (Table 2).

**Table 1:** Clinicopathological features of female cats with mammary carcinoma enrolled in this study (*n* = 115)

Clinicopathological feature	No. of animals (%)	Clinicopathological feature	No. of animals (%)
Age (Mean ± SD)	11.40 ± 2.82 years	HP classification	
<10 years	32 (27.8)	Papillary-cystic carcinoma	7 (6.1)
≥10 years	79 (68.7)	Cribriform carcinoma	10 (8.7)
Unknown	4 (3.5)	Mucinous carcinoma	11 (9.5)
		Solid carcinoma	20 (17.4)
		Tubular carcinoma	30 (26.1)
		Tubulopapillary carcinoma	37 (32.2)
Breed		Malignancy grade	
Not determined	95 (82.6)	I	3 (2.6)
Siamese	10 (8.7)	II	21 (18.3)
Persian	7 (6.1)	III	91 (79.1)
Norwegian Forest Cat	3 (2.6)		
Spayed		Necrosis	
No	59 (51.3)	No	30 (26.1)
Yes	52 (45.2)	Yes	85 (73.9)
Unknown	4 (3.5)		
Contraceptives		Lymphatic invasion	
No	35 (30.4)	No	91 (79.1)
Yes	53 (46.1)	Yes	24 (20.9)
Unknown	27 (23.5)		
Treatment		Lymphocytic infiltration	
Mastectomy	97 (84.3)	No	33 (28.7)
Mastectomy + Chemo	8 (7.0)	Yes	80 (69.6)
None	10 (8.7)	Unknown	2 (1.7)
Multiple mammary tumors		Tumor ulceration	
No	42 (36.5)	No	96 (83.5)
Yes	73 (63.5)	Yes	19 (16.5)
Disease stage (TNM)		Ki-67 index	
I	25 (21.7)	Low (<14%)	31 (26.9)
II	20 (17.4)	High (≥14%)	83 (72.2)
III	52 (45.2)	Unknown	1 (0.9)
IV	13 (11.3)		
Not determined	5 (4.4)		
Lymph node status		PR status	
Negative	61 (53.0)	Negative	66 (57.4)
Positive	44 (38.3)	Positive	49 (42.6)
Unknown	10 (8.7)		
Localization		ER status	
M1	17 (14.8)	Negative	86 (74.8)
M2	22 (19.1)	Positive	29 (25.2)
M3	43 (37.4)		
M4	26 (22.6)		
Unknown	7 (6.1)		
Recurrence		HER2 status	
No	39 (33.9)	Negative	83 (72.2)
Yes	64 (55.7)	Positive	32 (27.8)
Unknown	12 (10.4)		
Survival		Serum CXCL12 levels	
No	53 (46.1)	Negative (<2 ng/ml)	17 (14.8)
Yes	58 (50.4)	Positive (≥2 ng/ml)	25 (21.7)
Unknown	4 (3.5)	Not determined	73 (63.5)
Tumor size			
≤1 cm	21 (18.3)		
>1 cm	94 (81.7)		

*HP* Histopathological

**Table 2:** Biomarker status in regional and distant metastases collected from cats with mammary carcinoma

Regional Metastases			Distant Metastases		
Biomarker	Axillary LN (*n* = 18)	Retromammary LN (*n* = 29)	Biomarker	Lung (*n* = 20)	Liver (*n* = 7)
PR status			PR status		
Negative	8 (44.4)	16 (55.2)	Negative	14 (70.0)	6 (85.7)
Positive	10 (55.6)	13 (44.8)	Positive	6 (30.0)	1 (14.3)
ER status			ER status		
Negative	8 (44.4)	24 (82.8)	Negative	14 (70.0)	7 (100)
Positive	10 (55.6)	5 (17.2)	Positive	6 (30.0)	0 (0.0)
HER2			HER2		
Negative	13 (72.2)	20 (69.0)	Negative	20 (100)	7 (100)
Positive	5 (27.8)	9 (31.0)	Positive	0 (0.0)	0 (0.0)
Ki-67 index			Ki67 index		
Low (<14%)	2 (11.1)	4 (13.8)	Low (<14%)	7 (35.0)	2 (28.6)
High (≥14%)	16 (88.9)	25 (86.2)	High (≥14%)	13 (65.0)	5 (71.4)

*LN* Lymph nodes

### CXCR4 is highly expressed in FMC and significantly more in primary tumors than in metastases contrasting with its ligand CXCL12

**Fig. 1:**
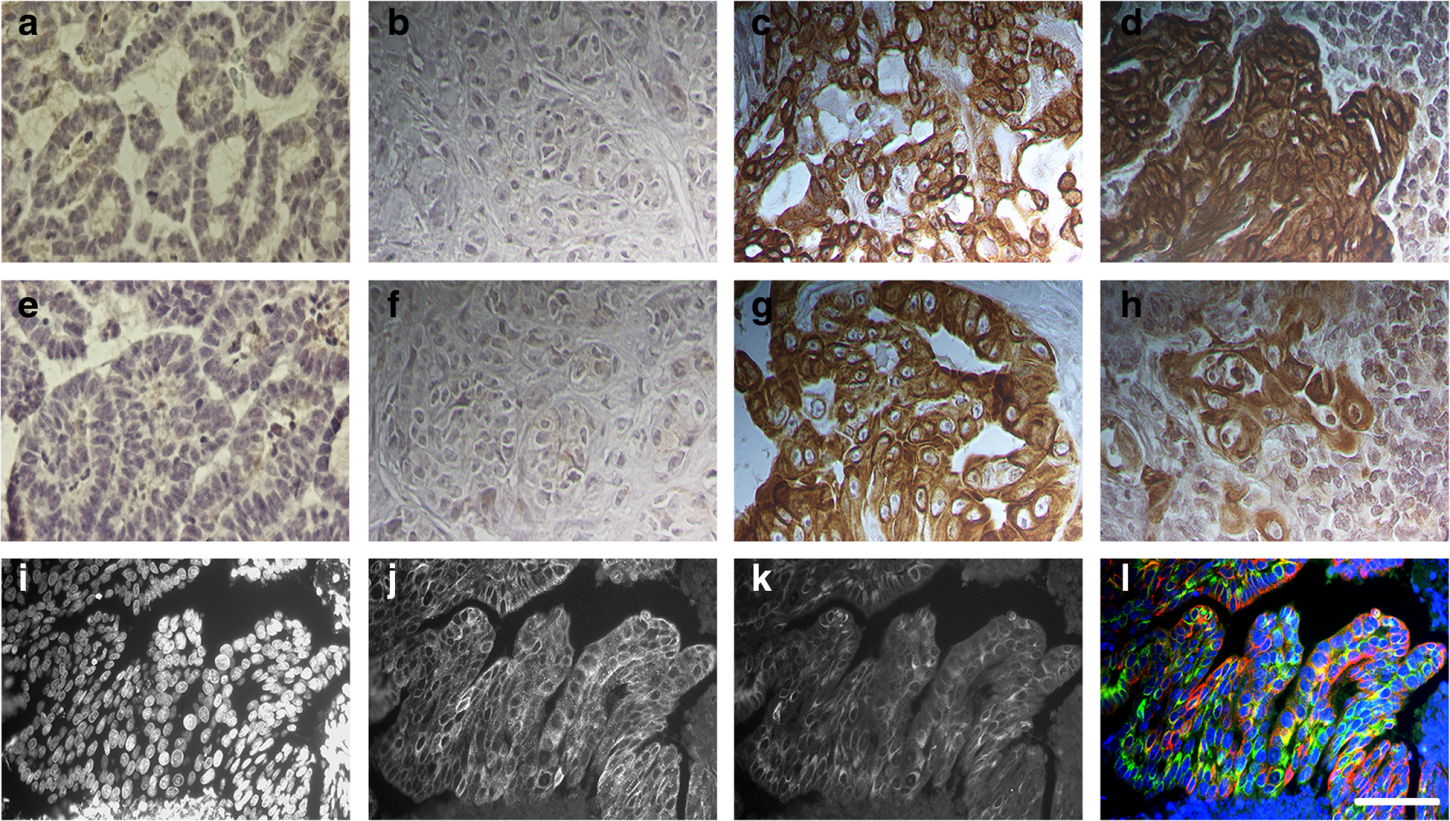
Immunohistochemical and immunofluorescence analysis of CXCR4 and CXCL12 expression in normal mammary tissue, in primary tumors (PT) and in metastatic lesions reflects the pathology of FMC. Photomicrographs (**a**, **b**, **e** and **f**) represent immunohistochemical control samples of normal tissue stained for CXCR4 (**a**) and CXCL12 (**e**) and PT in the absence of CXCR4 (**b**) and CXCL12 (**f**) antibody incubation. Photomicrographs (**c** and **d**) show representative samples of PT (**c**) and regional metastasis (RM) (**d**) with CXCR4 positive staining in cell membrane and cytoplasm. Photomicrographs (**g** and **h**) illustrate representative samples of PT (**g**) and RM (**h**) with CXCL12 positive staining observed mainly in the cytoplasm. Photomicrograph (**l**) represents immunolabeling of PT cells in appropriate fluorescence filter sets, corresponding to the signals of DAPI (**i**), CXCR4-Alexa Fluor® 594 (**j**) and CXCL12-Alexa Fluor® 488 (**k**). All photomicrographs were taken in the high-powered magnification, × 400 and the scale bar represents 40 μm

The CXCR4 and CXCL12 expression analyzed by immunohistochemistry, was evaluated using a semi-quantitative system previously published [6, 8, 9, 23, 29–31]. The expression of CXCR4 was mostly confined to the cytoplasmic membrane and cytoplasm of neoplastic cells (Fig. 1c, d, j) whereas CXCL12 was mainly located to cytoplasm and, in a lesser extend to cytoplasmic membrane of tumor cells (Fig. 1g, h, l) but also to tumor-associated macrophages and cancer-associated fibroblasts. Although both proteins are highly expressed in the majority of PT and metastases, high variability in extension and in staining intensity was detected in the tumor samples. Normal mammary tissue did not presented CXCR4 (Fig. 1a) or CXCL12 staining (Fig. 1e), as well, as control PT in the absence of CXCR4 (Fig. 1b) or CXCL12 (Fig. 1f) antibody incubation. As expected, double immunofluorescence labeling confirmed the distribution patterns of CXCR4 and CXCL12 (Fig. 1i, j, k, l). The expression in PT was increased in 93 out of 113 queens (82.3%) for CXCR4 and in 89 out of 114 (78.1%) for CXCL12. In RM, 34 out of 48 samples (70.8%), collected from 47 animals stained positive for CXCR4 while 47 out of 49 samples (95.9%) showed positive staining for the CXCL12 ligand. Finally, 17 out of 31 (54.8%) DM, collected from 20 queens, showed CXCR4 positivity, whereas, CXCL12 expression was observed in all samples (Table 3). On the other hand, the expression rate of CXCR4 decreased from PT to distant metastasis in 27.5% while an opposite trend is observed for CXCL12 expression, with an increased expression of 21.9% from PT (78.1%) to metastasis (100.00%) (Table 3). These differences were significant for CXCR4 expression rates between PT and metastases (*p* = 0.0067; OR = 2.55; 95% CI: 1.31–4.98) and also for CXCL12 status (*p* < 0.0001; OR = 10.96; 95% CI: 25.13–47.76). Indeed, 76.3% of the cats with CXCR4 positive PT (29/39), showed RM CXCR4 positive and only 50.0% displaying CXCR4 positive DM (7/14). Sixty percent (3/5) and 75.0% (3/4) of CXCR4-negative PT became CXCR4 positive in RM and DM, respectively. The majority of the CXCL12 positive PT, conserved the CXCL12 expression in RM (36/37 animals; 97.3%) and DM (13/13; 100%) while most of CXCL12 negative PT became positive in RM (7/8, 87.5%) and DM (7/7; 100%). No significant associations were found between CXCR4 and CXCL12 expression in PT and metastases, and clinical and pathological features.

**Table 3:**
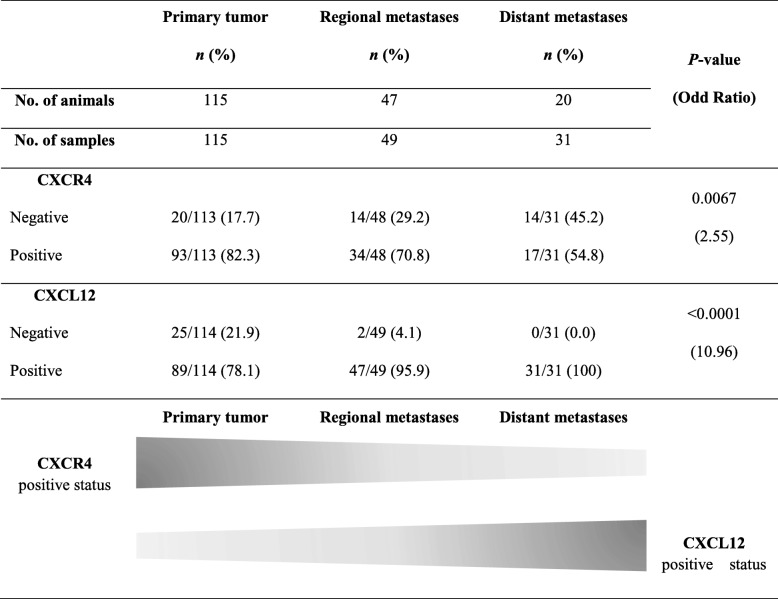
CXCR4 and CXCL12 status in primary tumors, regional and distant metastases of cats with mammary carcinoma

### CXCR4 and CXCL12 tumor status contributes to serum CXCL12 levels

**Fig. 2:**
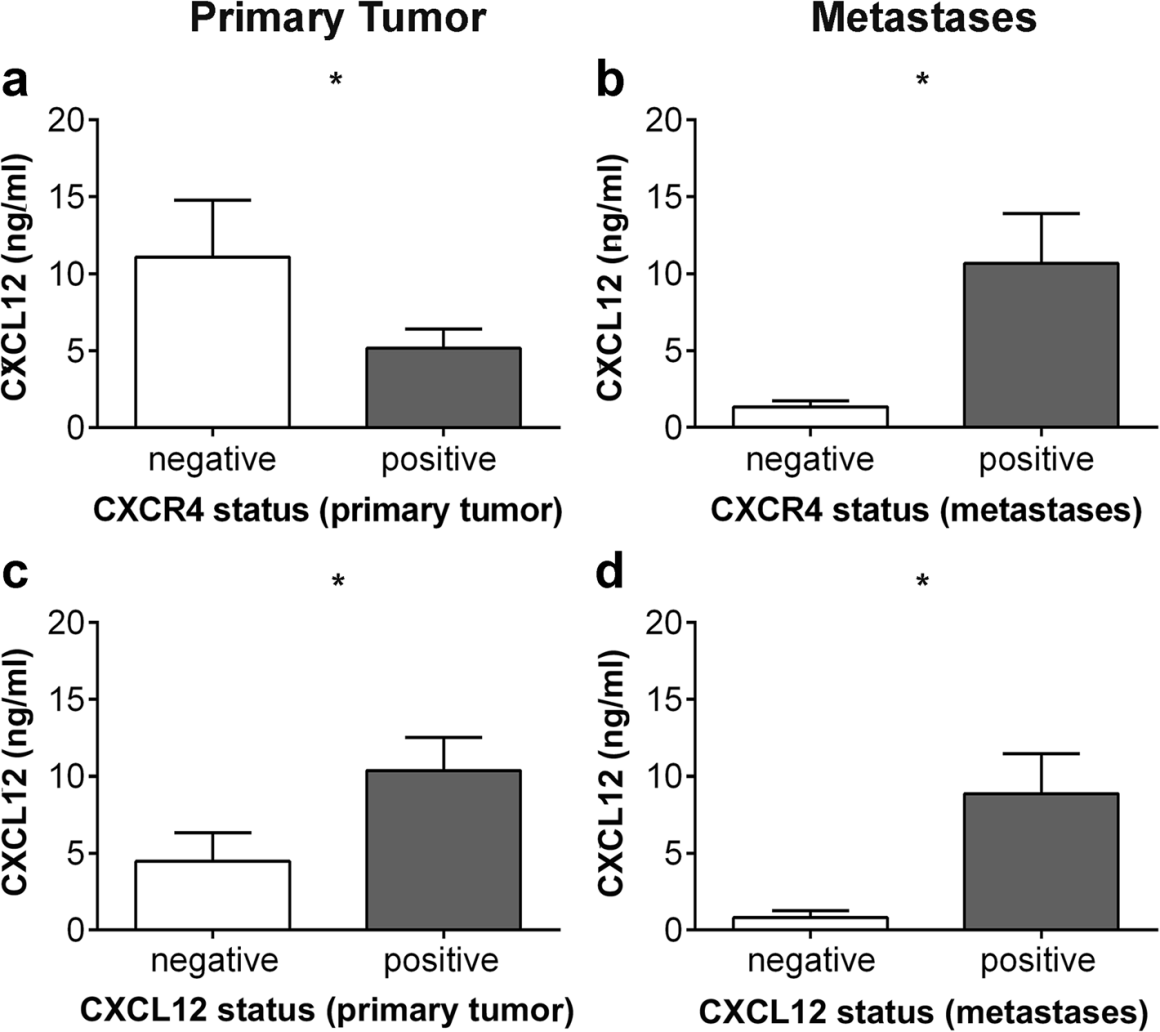
CXCR4 and CXCL12 expression in primary tumors (PT) and metastases significantly contribute to serum CXCL12 concentration. CXCL12 levels were quantified in blood serum by ELISA while the expression of CXCR4 in PT (**a**) and metastases (**b**) and of CXCL12 in PT (**c**) and metastases (**d**), was assessed by immunohistochemistry. The non-parametric Mann-Whitney was used to compare serum CXCL12 levels between the cats having CXCR4/CXCL12-negative tumors and cats with CXCR4/CXCL12-positive tumors. Bars represent the mean value ± SEM and * indicates a significant difference

Cats with CXCR4-negative PT presented significant higher serum CXCL12 levels (11.06 ± 3.72 ng/ml) (*p* = 0.0324) compared with serum CXCL12 values from cats presenting CXCR4 positive tumors (5.16 ± 1.26 ng/ml) (Fig. 2a). A significant opposite condition was observed for CXCL12 status in PT, with CXCL12-negative tumors presenting significant lower (*p* = 0.0277) blood serum CXCL12 levels (4.48 ± 1.86 ng/ml) than CXCL12-positive tumors (10.36 ± 2.16 ng/ml) (Fig. 2b). The same significant pattern was observed in CXCL12 metastatic tumors, where CXCL12-negative metastases were significant associated (*p* = 0.0310) with low CXCL12 serum levels (0.82 ± 0.44 ng/ml), comparing with CXCL12-positive metastasis (8.88 ± 2.57 ng/ml) (Fig. 2d). Contrasting to what was obtained for CXCR4 in PT, high serum CXCL12 levels (10.68 ± 3.22 ng/ml) were significant correlated with CXCR4 positive status in metastases, whereas CXCR4-negative metastases presented significant lower (*p* = 0.0341) serum CXCL12 levels (1.34 ± 0.38 ng/ml) (Fig. 2c).

### HER2-overexpressing FMC presented increasing CXCR4 expression from PT to metastases and CXCL12 status had a prognostic value

**Fig. 3:**
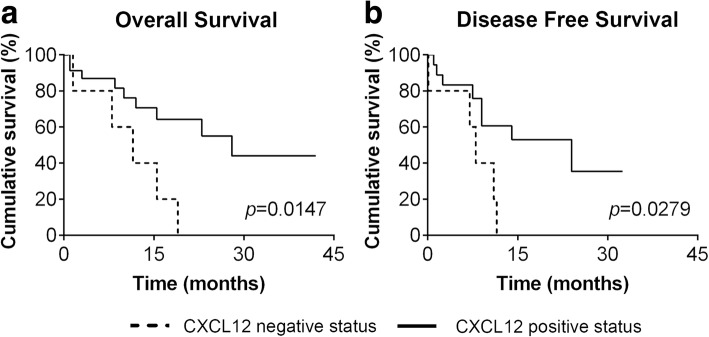
CXCL12 status has prognostic value in cats with HER2-overexpressing mammary carcinoma. **a** Kaplan-Meier analysis showing the overall survival (OS) of cats with HER2-overexpressing mammary carcinoma, presenting CXCL12 positive and negative expression in primary tumors (PT). **b** Kaplan-Meier curve illustrating the disease-free survival (DFS) of cats with HER2-overexpressing mammary carcinoma, stratified according to CXCL12 expression in PT. Comparison between the two groups was performed by the log-rank (Mantel Cox) test

The CXCR4 and CXCL12 expression, analyzed by IHC, was also evaluated according to their PT molecular subtype (Table 4). Luminal A (LA), luminal B (LB), normal and basal triple-negative (TN) tumors mimicked the trend of CXCR4 and CXCL12 expression previous described in PT, RM and DM of the total cohort of cats with mammary carcinoma. However, cats with HER2-overexpressing tumors (LB-HER2/HER2) showed a different pattern. In this group of animals, the CXCR4 expression rate was significant higher in distant metastases (100%) and regional metastases (84.6%) than in PT (77.4%) (*p* = 0.012; OR = 6.40; 95% CI: 1.34–30.62), with cats with CXCR4 positive mammary carcinomas showing CXCR4 positive status in the majority of RM (10/11, 90.9%) and in all DM (4/4, 100%). Finally, significant differences in OS (*p* = 0.0147) and DFS (*p* = 0.0279) rates were found between animals with HER2-overexpressing mammary carcinoma showing positive CXCL12 status and cats exhibiting negative CXCL12 expression (Fig. 3a, b). In these animals, CXCL12 positive expression was associated with increased OS and DFS.

**Table 4:** CXCR4 and CXCL12 status in primary tumors, regional and distant metastases of cats with mammary carcinoma, stratified accordingly to the molecular subtype of the primary tumor

		Primary tumor	Regional metastases	Distant metastases
Luminal A	CXCR4			
	Negative	1/13 (7.7)	2/4 (50.0)	2/3 (66.7)
	Positive	12/13 (92.3)	2/4 (50.0)	1/3 (33.3)
	CXCL12			
	Negative	5/13 (38.5)	0/4 (0.00)	0/3 (0.00)
	Positive	8/13 (61.5)	4/4 (100)	3/3 (100)
Luminal B	CXCR4			
	Negative	3/31 (9.7)	5/15 (33.3)	3/4 (75.0)
	Positive	28/31 (90.3)	10/15 (66.7)	1/4 (25.0)
	CXCL12			
	Negative	8/32 (25.0)	0/15 (0.00)	0/4 (0%)
	Positive	24/32 (75.0)	15/15 (100)	4/4 (100)
Luminal B-HER2+/HER2+	CXCR4			
	Negative	7/31 (22.6)	2/13 (15.4)	0/5 (0.0)
	Positive	24/31 (77.4)	11/13 (84.6)	5/5 (100)
	CXCL12			
	Negative	6/30 (20.0)	2/14 (14.3)	0/5 (0.0)
	Positive	24/30 (80.0)	12/14 (85.7)	5/5 (100)
Triple negative	CXCR4			
	Negative	9/38 (23.7)	5/15 (33.3)	7/13 (53.9)
	Positive	29/38 (76.3)	10/15 (66.7)	6/13 (46.1)
	CXCL12			
	Negative	6/39 (15.4)	0/15 (0.0)	0/13 (0.00)
	Positive	33/39 (84.6)	15/15 (100)	13/13 (100)

*HER2+* HER2-overexpressing subtype

## Discussion

Based in many in vitro and in vivo evidences, nowadays, it is widely accepted that CXCR4/CXCL12 axis is involved in tumor growth, migration and invasion of human breast tumor cells [1, 2, 10, 11]. However, its specific role is still a controversial issue, probably due to the tumor heterogeneity but also because of the different techniques, methodologies and protocols used in quantification of CXCR4 and CXCL12 expression. On the other hand, the information regarding CXCR4/CXCL12 axis during the development of FMC is almost absent, despite the fact FMC being considered as suitable cancer model [17–19]. Recently our group identified CXCL12 as a reliable blood serum marker for FMC, being predominantly relevant for HER2-overexpressing tumors diagnosis [24]. In order to provide a deeper insight into the role of the CXCR4/CXCL12 axis in FMC, CXCR4 and CXCL12 expression was analyzed and quantified in PT, RM and DM from female cats with mammary carcinoma and its expression was correlated with blood serum CXCL12 levels and molecular subtypes. As in woman breast tissues, where CXCR4 protein is highly expressed in ductal carcinoma, progressively increasing from atypical hyperplasia to carcinoma and absent or expressed in very low levels in normal breast epithelium [9, 29, 34], normal feline mammary tissue used in this study revealed no CXCR4 expression. In accordance to our result, the only study that evaluated CXCR4 at protein level by IHC in FMC also showed weakly positive expression in epithelial cells of benign tumors and no detectable CXCR4 expression in normal mammary tissue [23]. Moreover, FMC tissues also expressed more CXCR4 mRNA than normal mammary tissue [21, 22]. Many studies do not report the protein expression pattern; nevertheless in our study immunoreactivity to CXCR4 was observed mainly in the cell membrane while CXCL12 appears more often in the cytoplasm of primary and metastatic tumor cells. Indeed, Ferrari et al., observed that the expression of CXCR4 was mostly limited to the membrane and cytoplasm of feline neoplastic cells in a significant high number of samples (29/31 samples, 93%) [23]. As in our study, expression differed in intensity and staining pattern but we obtained 82% of positive PT (93/113 animals). In human breast primary tumors, heterogeneity of expression pattern is often found and the positivity rate, is highly diverse [29, 30, 35–42]. This variability can be explained by the different IHC and scoring methods but also by the diversity of CXCR4 primary antibodies used. Cytoplasmic CXCL12 immunostaining of primary breast cancer cells was already been showed before [9, 31, 40, 43, 44] and may reflect endogenous CXCL12 being processed just before secretion, as chemokine cell storage is uncommon. On the other hand, membrane CXCL12 immunostaining is probably due to the binding of exogenous protein to its main receptor CXCR4. Immunofluorescence and IHC have shown that CXCL12 is expressed most likely in the tumor cells and expression rates of 70.9% [44], 66.8% [31] or 70.6% [43] in breast tumors are relatively close to the CXCL12 expression obtained for feline mammary tumors in this study (78.1%). Even though not accountable for the CXCL12 scoring, we also found positive CXCL12 expression in tumor-associated macrophages and cancer-associated fibroblasts, in accordance with previous studies where CXCL12 is mainly secreted by tumor cells, but also by stromal cells, including tumor-associated macrophages and cancer-associated fibroblasts [7, 29]. Although feline CXCR4 and CXCL12 were also highly expressed in regional and distant metastases, we observed a significant decrease in CXCR4 and an increase in CXCL12 expression from PT to DM. Moreover, half of the animals that presented CXCR4 positive PT, did not preserve its positivity in DM, whereas CXCL12 was upregulated in 100% of DM that were CXCL12 negative in PT. Indeed, CXCR4 expression in PT promotes metastasis of CXCR4 positive tumor cells to sites commonly affected by metastatic breast cancer (lymph node, lung, liver, bone marrow and brain) where its ligand CXCL12 is generated in large quantity [5]. Therefore it is expected that feline CXCR4 will be more expressed in PT while CXCL12 is being constitutively expressed in liver and lung. Moreover, immunofluorescence and flow cytometry analysis of orthotopic primary breast tumors also presented a remarkably higher expression level of CXCR4, in contrast to lung metastatic lesions [45]. The researchers suggest that CXCR4 is downregulated in metastasized cancer cells depending on the favorable change of the environment that induces dormancy to cancer cells, although not dependent on the expression level of CXCL12 in lung tissue [45] In addition, lower levels of CXCL12 in PT were also observed in human breast cancer. Reduced CXCL12 expression was due to hyper methylation in the CXCL12 promoter region [38], as CXCL12 produced in PT can limit cell migration potential by desensitizing cells to endocrine ligand, or alternatively, by nullifying chemokine gradients produced in distant tissues [46]. Furthermore, downregulation of CXCL12 expression by mesenchymal stromal cells in PT and upregulation of TGF-β and CXCR7, promotes breast cancer cell metastasis to the lungs [47]. Next, we aimed to understand whether tumor expression of CXCR4 and CXCL12 may significantly contribute to serum CXCL12 levels. The results confirm our recent study where we found that cats with CXCR4 positive PT exhibit significantly lower serum CXCL12 levels than cats with CXCR4-negative mammary carcinomas [24]. Indeed, in breast cancer patients it was reported that low serum CXCL12 levels may favor the migration of tumor cells overexpressing CXCR4, predicting and promoting the development of distant metastases [9, 48]. Considering that feline CXCR4 expression decreases at metastatic sites, it is also reasonable to accept the association of low serum CXCL12 levels with lesser CXCR4 expression in metastatic lesions. On the other hand, a significant strong correlation was obtained between CXCL12 expression in PT and metastasis and CXCL12 serum levels. Although the cellular source of CXCL12 levels could be highly diverse [49], secretion of CXCL12 by CXCL12 positive tumor cells may account for higher serum concentrations of CXCL12. Altogether, these data highlight the impact of feline CXCR4 and CXCL12 expression in PT and metastases on serum CXCL12 levels uncovering this new component on the CXCR4/CXCL12 axis, during FMC. Next, the rates of CXCR4 and CXCL12 expression in PT, RM and DM were evaluated according to PT molecular subtype. We verified that the pattern of CXCL12 in all tumor subtypes was the same as the one described for all the cohort, with an increased trend of expression from PT to distant metastases. The decreased trend of CXCR4 expression from PT to DM observed for the entire cohort was also maintained in the luminal A, luminal B and TN tumor subtypes. However, in HER2-overexpressing tumors, CXCR4 expression raised up from PT to DM. At metastatic lesions, HER2-overexpressing tumors presented significant higher CXCR4 expression than the other tumor molecular subtype. There have been few attempts to correlate CXCR4 with different molecular subtypes in human breast cancer. However, these studies regularly take into account PT expression. One of the first studies established a functional link between the HER2 and CXCR4 signaling pathway PI3K/Akt/mTOR, responsible for the HER2-induced CXCR4 expression in PT [50]. The researchers also demonstrate that HER2 is involved in the inhibition of the CXCL12-induced CXCR4 ubiquitination [50]. Additionally, significant increase of high CXCR4 and HER2 co-expression was observed in tumors with extensive lymph node metastases [36]. Because an intrinsic positive link between both proteins has been established in PT and correlated with metastasis, it is suitable to admit that the feline CXCR4 positive PT cells induced by HER2, are the ones related to the high expression levels of CXCR4 in metastatic lesions. Recently, it was found that pomolic acid suppresses HER2 and CXCR4 expression in HER2-positive breast cancer cells through ERK pathway and NF-κB inactivation [51] and CXCR4 inhibitors efficiently reduces tumor growth and metastasis in both Herceptin-sensitive and Herceptin-resistant HER2 patient-derived xenografts thus CXCR4 being considered a very promising therapeutic target in patients with HER2-overexpressing breast cancer [16]. Among HER2-overexpressing cats, there was a significant difference in OS and DFS curves between the positive and negative CXCL12 tumor expression groups, with CXCL12 negative PT associated with unfavorable prognosis. In breast tumors this fact has been observed only in non-basal and ER-positive tumors [31, 43]. Nevertheless, poor prognosis could be justified in HER2-overexpressing tumors as it seems that reduced CXCL12 negative primary tumor cells are in a better position to receive endocrine CXCL12 signals, promoting their migration towards ectopic sources of the ligand through a chemotactic gradient, together with HER2 upregulation of CXCR4 tumor cells that could metastasize to distant organs.

In summary, our results showed a different CXCR4 and CXCL12 pattern of expression in PT and metastases of cats with mammary carcinoma. While feline CXCR4 is significantly more expressed in PT, CXCL12 is highly abundant in distant metastatic organs such as liver and lung, as it has been observed for woman breast cancer. Moreover, CXCR4 and CXCL12 tumor expression significantly contribute to serum CXCL12 concentrations. However, there is some differences in the impact of both these proteins in cats with different molecular subtypes. Indeed, in HER2-overexpressing tumors, it is observed a HER2-dependent CXCR4 upregulation and a better prognosis mediated by CXCL12 ovexpression.

## Conclusion

The results of the present study uncover part of the complex interaction between CXCR4 and its ligand in PT but also in metastases of FMC. Although our findings will require a deeper insight into the regulatory pathways involved in the CXCR4/CXCL12 axis, the present study highlights the axis as an important target for future therapy in FMC as it has been proved to be in human breast cancer. This work also emphasizes FMC as a suitable spontaneous cancer model which may allow to predict novel therapeutic strategies in cats and in humans.

## Abbreviations

BSA: Bovine serum albumin
CXCL12: Chemokine ligand 12
CXCR4: C-X-C Chemokine Receptor Type 4
CXCR7: C-X-C Chemokine Receptor Type 7
DFS: Disease free-survival
DM: Distant metastases
ELISA: Enzyme-linked immunosorbent assay
ER: Estrogen receptor
ERK1/2: Extracellular signal-regulated kinase 1/2
FMC: Feline mammary carcinoma
HER2: Human epidermal growth factor receptor type II
HRP: Horseradish peroxidase
IHC: Immunohistochemistry
LA: Luminal A
LB: Luminal B
MAPK: Mitogen-activated protein kinase
min: minutes
NF-κB: Nuclear factor kappa light-chain-enhancer of activated B cells
OR: Odd ratio
OS: Overall survival
PBS: Phosphate buffer solution
PI3K: Phosphoinositide 3-kinase
PR: Progesterone receptor
RM: Regional metastases
SDF-1: Stromal cell-derived-factor-1
SEM: Standard error of mean
TGF-β: Transforming growth factor-beta
TN: Triple negative
